# Impact of a Histone Deacetylase Inhibitor—Trichostatin A on Neurogenesis after Hypoxia-Ischemia in Immature Rats

**DOI:** 10.3390/ijms21113808

**Published:** 2020-05-27

**Authors:** Teresa Zalewska, Joanna Jaworska, Joanna Sypecka, Malgorzata Ziemka-Nalecz

**Affiliations:** NeuroRepair Department, Mossakowski Medical Research Centre, Polish Academy of Sciences, 02-106 Warsaw, Poland; terezal@imdik.pan.pl (T.Z.); jjaworska@imdik.pan.pl (J.J.); jsypecka@imdik.pan.pl (J.S.)

**Keywords:** neonatal hypoxia-ischemia, neurogenesis, BDNF-TrkB pathway, CREB

## Abstract

Hypoxia-ischemia (HI) in the neonatal brain frequently results in neurologic impairments, including cognitive disability. Unfortunately, there are currently no known treatment options to minimize ischemia-induced neural damage. We previously showed the neuroprotective/neurogenic potential of a histone deacetylase inhibitor (HDACi), sodium butyrate (SB), in a neonatal HI rat pup model. The aim of the present study was to examine the capacity of another HDACi—Trichostatin A (TSA)—to stimulate neurogenesis in the subgranular zone of the hippocampus. We also assessed some of the cellular/molecular processes that could be involved in the action of TSA, including the expression of neurotrophic factors (glial cell line-derived neurotrophic factor (GDNF), nerve growth factor (NGF), and brain-derived neurotrophic factor (BDNF)) as well as the TrkB receptor and its downstream signalling substrate— cAMP response element-binding protein (CREB). Seven-day-old rat pups were subjected to unilateral carotid artery ligation followed by hypoxia for 1 h. TSA was administered directly after the insult (0.2 mg/kg body weight). The study demonstrated that treatment with TSA restored the reduced by hypoxia-ischemia number of immature neurons (neuroblasts, BrdU/DCX-positive) as well as the number of oligodendrocyte progenitors (BrdU/NG2+) in the dentate gyrus of the ipsilateral damaged hemisphere. However, new generated cells did not develop the more mature phenotypes. Moreover, the administration of TSA stimulated the expression of BDNF and increased the activation of the TrkB receptor. These results suggest that BDNF-TrkB signalling pathways may contribute to the effects of TSA after neonatal hypoxic-ischemic injury.

## 1. Introduction

Neonatal hypoxia-ischemia (HI) insults due to perinatal asphyxia remain the main cause of neurological injury resulting from birth complications. They are caused by the restriction of blood supply and, as a consequence, the deficiency of oxygen and glucose in the brain. The incidence of HI injury occurs in 1.5–2.5 of a 1000 live term births [1]. Although the notable progress in intensive care has improved the survival of infants suffering from HI, still 40%–60% of babies affected by neonatal asphyxia die or develop neurological dysfunctions such as intellectual disability, motor impairment, epilepsy and spastic paresis [1]. Certainly, brain damage following hypoxic-ischemic insults is a process that evolves over several hours to days and hence it provides a possibility for therapeutic intervention in the HI-induced cascade of intracellular events. Until this point, the treatment and prevention options have been limited. At present, therapeutic hypothermia is only one accepted neuroprotective treatment for term babies affected by this disease. Clinically, such hypothermia is only partially effective [2,3]. Thus, there is a considerable interest in developing a complementary strategy, such as regenerative medicine using stem cells. Although neurogenesis after neonatal HI has been documented [4], it was further found that the capacity for endogenous regeneration seems to be limited and insufficient for replacing the lost neurons [5]. Hence, the premature brain is not capable of completely regenerating itself, due to the extensive cellular degeneration and to the disturbed expression of differentiation factors present usually in neurogenic areas in physiological conditions. A major challenge in this field is overcoming the redundancy of the multiple pathways activated in response to injury using a single intervention that will provide an efficient stimulation of brain neurogenesis [6]. 

Several studies using various models of brain ischemia in adult rodents have employed epigenetic modifications that control transcription processes such as histone lysine acetylation/deacetylation [7] in stroke-induced brain damage and recovery [8,9,10]. The epigenetic processes are under the control of two different classes of enzymes: histone deacetylases (HDACs) and acetyltransferases (HATs). The resulting changes in chromatin organization other than the DNA sequence can influence the expression of various gene pathways that regulate stress responses, cell survival and neurogenesis [11]. It has been documented that the administration of compounds that increase the lysine acetylation of H3 and H4 histones by hindering histone deacetylases (HDACs), such as, e.g., sodium butyrate (SB), Trichostatin A (TSA), and valproic acid (VPA), reduced brain injury, enhanced neurogenesis and increased functional performance in adult rodents subjected to experimental ischemia [12,13,14,15,16]. Although the acetylation of histones is a crucial post-translational modification of proteins accountable for the governing of pivotal intracellular pathways, histone deacetylases inhibitors (HDACis) equally modified the activity of several non-histone proteins, such as transcription factors, signal transduction mediators, chaperone proteins, hormone receptors, and cytoskeletal proteins [17]. Altogether, these results provided evidence that HDAC inhibitors, primarily SB, could be a promising tool for the treatment of ischemia. A few available reports suggest a beneficial effect of histone deacetylase inhibitors also in the immature brain [18,19,20,21]. In line with these findings, our previous data revealed that treatment with HDAC inhibitor—sodium butyrate (SB)—applied directly after the induction of hypoxia-ischemia to 7-day-old rats demonstrates a neuroprotective/neurogenic effect in the subgranular zone of the hippocampus (SGZ) as well as in the subventricular (SVZ) area [22,23].

Little is known about the beneficial role of structurally different histone deacetylase inhibitors (HDACi) in neonatal brain injury. It may be thought that chemical structure determines the mechanism of action and the exhibition of some HDACs isoforms specificity, which may differ between adult and immature animals. The knowledge about, e.g., Trichostatin A, a hydroxamic acid-based inhibitor acting as a protective factor, initially comes from studies in ischemic adult rodents [13]. Only one report described the impact of TSA on oligodendrocyte development in the lipopolysaccharide (LPS)-sensitized neonatal HI injury. However, the mechanism causing the neuroprotection by the TSA in female neonates was different from the mechanisms reported in adult animals [20]. 

Therefore, in the present study we aimed to examine the effect of Trichostatin A treatment on neurogenesis and gliogenesis in neonatal model of hypoxia-ischemia (HI). We also studied the molecular mechanism triggered by this inhibitor in the immature brain. It is known that, in response to HI, the damaged cells release an array of factors, some of which are inhibitory, whereas some are stimulatory for neurogenesis. Among these factors, growth factors, in conjunction with their receptors, have been widely accepted as important mediators for the generation and survival of newborn cells in the developing nervous system. Furthermore, they are involved in the regeneration of nerve tissue after ischemia damage [24,25,26,27]. Therefore, we sought to determine the effect of TSA on growth factor molecules expression—Brain-derived neurotrophic factor (BDNF), Nerve growth factor (NGF) and Glial cell line-derived neurotrophic factor (GDNF), as well as the TrkB receptor and transcription factor cAMP response element-binding protein (CREB), which is activated downstream of BDNF-TrkB signalling pathway, after neonatal HI. In addition, we also investigated the potential participation of TSA in the acetylation of histone H3 and one non-histone target—alpha tubulin.

## 2. Results

### 2.1. The Effect of TSA on the Acetylation of Histone 3 and Alpha-Tubulin after Hypoxia-Ischemia 

**Figure 1:**
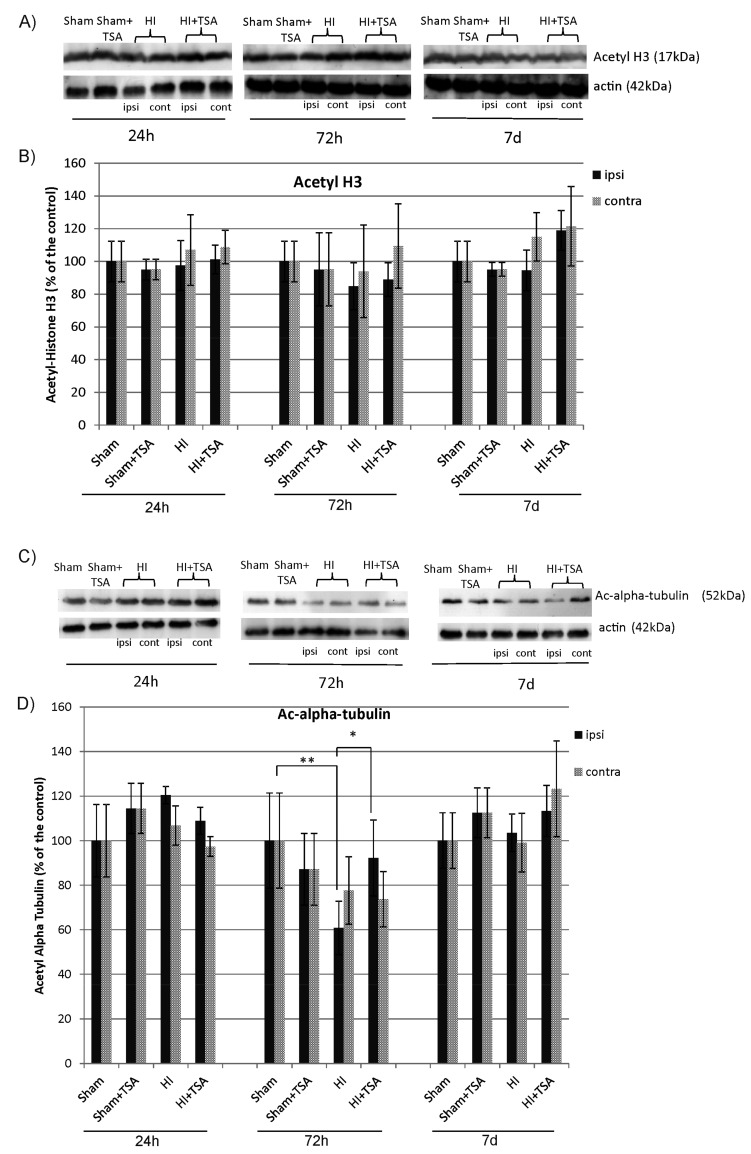
Effect of Trichostatin A (TSA) on the acetylation of Histone 3 (H3) and alpha tubulin after neonatal hypoxia-ischemia. (**A**,**C**) Representative immunoblots of acetylated H3 and alpha tubulin 24 h, 72 h and 7 days after HI, analyzed in experimental groups: sham-control (Sham), TSA-treated sham-control (Sham+TSA), hypoxia-ischemia (HI), TSA-treated hypoxia-ischemia (HI+TSA). The intensity of each band was quantified and normalized in relation to actin. (**B**,**D**) The graphs show the statistical analysis of densitometric data presented as a percent of the control value from indicated experimental groups. The values are mean ± SD from five animals per group and time point. Note the increased level of acetyl-alpha tubulin in ipsilateral hemisphere 72h after HI in TSA-treated rats compared to non-treated animals. The two-way ANOVA test indicates significant differences * *p* < 0.05 (effect of TSA treatment); ** *p* < 0.01 (effect of ischemia insult); Abbreviations: ipsi—ipsilateral, contra—contralateral.

At first, we determined the influence of TSA application on the acetylation of two inhibitor substrates—histone 3 (H3) and alpha-tubulin. Analysis of the Western Blot data clearly shows that the treatment with TSA did not change the level of acetylated histone H3 estimated in rat brain hemispheres at 1, 3, and 7 days after HI (Figure 1A,B). In contrast, the noticeable effect of TSA treatment on acetylation was observed in the case of the non-histone substrate—alpha tubulin. It was found that, 3 days after the insult, the level of acetylated alpha-tubulin in ipsilateral hemisphere decreased to 60.8% of sham-control (*p* < 0.01). The administration of TSA returned the degree of immunoreactivity to the control level (*p* < 0.05; HI vs. HI+TSA) (Figure 1C,D). 

### 2.2. Phenotypic Characterization of Proliferating Cells after Neonatal Hypoxic/Ischemia

**Figure 2:**
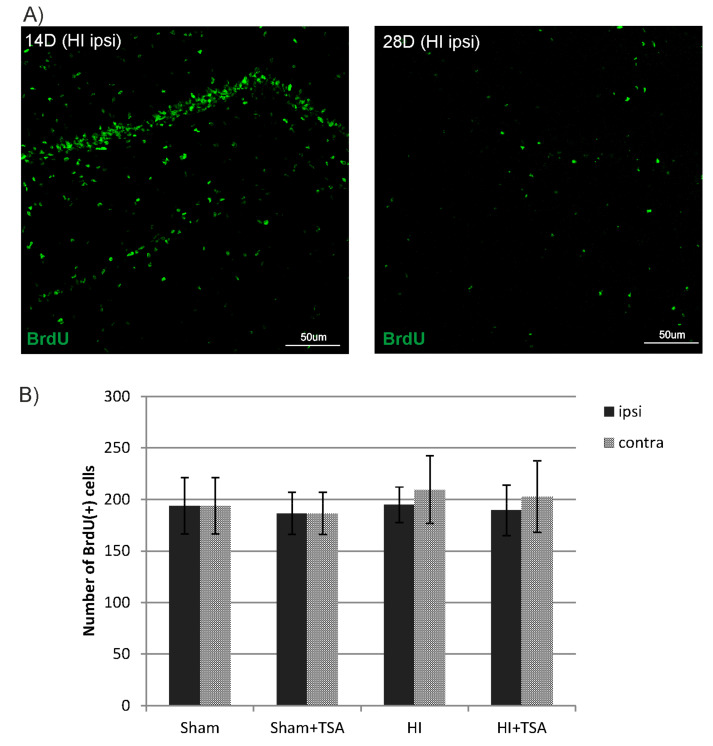
TSA has no effect on cell proliferation in the subgranular zone of the hippocampus (SGZ) after hypoxia-ischemia. (**A**) The confocal photomicrographs show newly divided cells (BrdU-positive) in ipsilateral DG 14 and 28 days after HI (D14 and D28). (**B**) The graph shows the number of BrdU-labeled nuclei within the DG of sham-control and HI animals (D14) with or without TSA treatment. The values are the mean ± SD of five animals per experimental group. The two-way ANOVA test did not indicate significant differences in the number of BrdU-labeled nuclei within the DG area in all investigated groups.

During the first step of these studies, we examined if the treatment with TSA affected cell proliferation in the hippocampal dentus gyrus (DG). The number of newly generated cells was assessed in the entire SGZ by monitoring the incorporation of BrdU. Rats received an injection of BrdU 4–6 days after HI and were sacrificed at 14 and 28 days after the insult. Our analysis indicates that BrdU immunoreactivity in the investigated brain area was more pronounced at 14 day after HI (D14) in all evaluated animals. Unexpectedly, neither HI alone nor HI with TSA treatment affected the pattern of cell proliferation. Thus, their numbers remained similar between hemispheres of given animals (Figure 2). 

**Figure 3:**
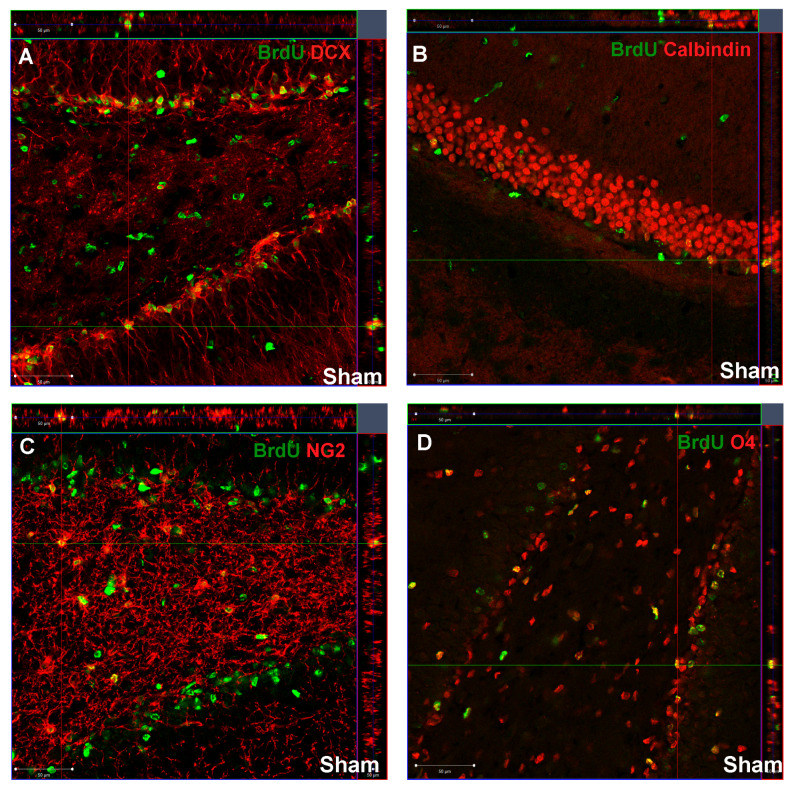
Newly divided cells in the DG of sham-control animals. The confocal photomicrographs show double-labeled newly generated: (**A**) neuroblasts (DCX(+)/BrdU(+)); (**B**) mature neurons (calbindin(+)/BrdU(+)); (**C**) oligodendrocyte progenitor cells (NG2(+)/BrdU(+)); (**D**) oligodendrocytes (O4(+)/BrdU(+)); in the SGZ of sham-control rats at postnatal day 21 (P21). Scale bar 50 µm.

To further characterize the fate of cells that incorporated BrdU, we used double staining with different neural antigens: DCX (immature neurons—neuroblasts), calbindin (mature neurons), NG2 (oligodendrocyte progenitor cells) and O4 (immature, non-myelinating oligodendrocytes). Double-labeled cells were counted in a clearly defined region of all groups. Figure 3 represents confocal photomicrographs (z-stacks) that demonstrate the co-localization of BrdU and cell markers in control DG. 

**Figure 4:**
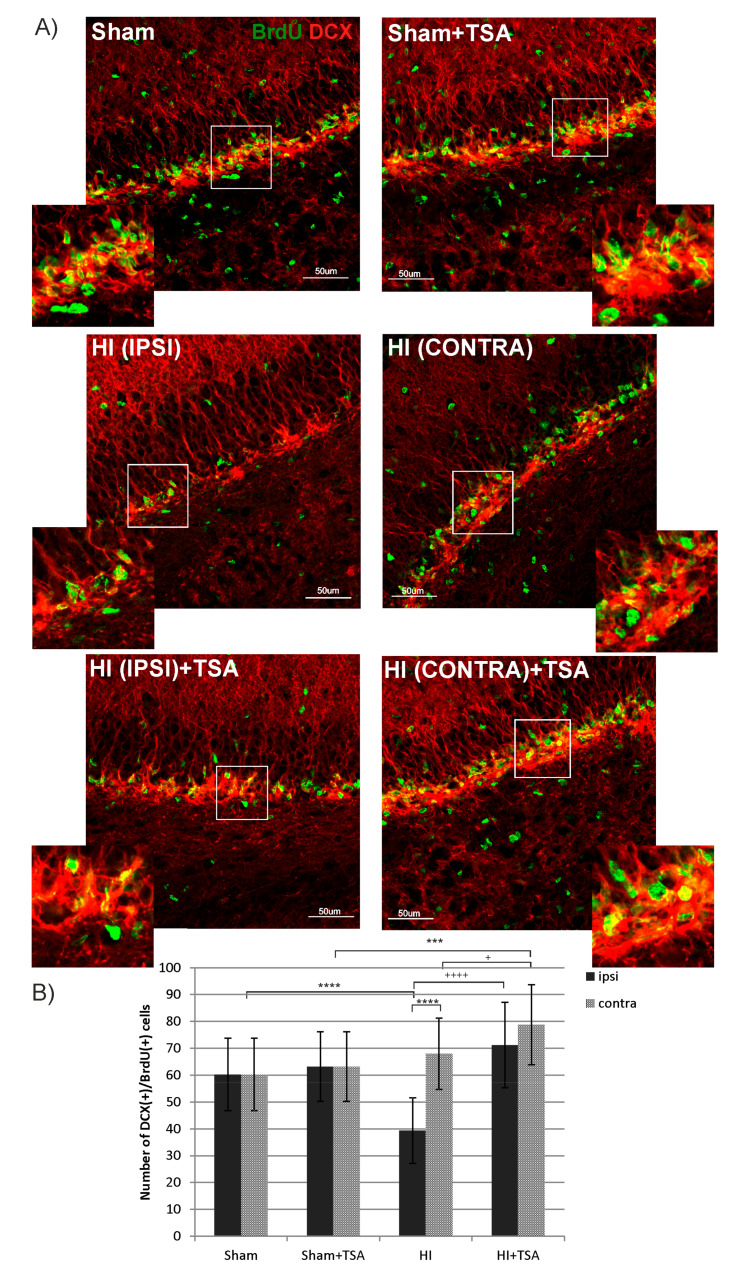
TSA increases the number of newborn neuroblasts in the SGZ after neonatal hypoxia-ischemia. (**A**) The confocal photomicrographs show double-labeled newly generated neuroblasts (DCX-positive) in the SGZ of sham-control and HI animals D14 with or without TSA treatment. Enlargements present areas marked in rectangles. Scale bar 50 µm. (**B**) The graph presents the number of BrdU/DCX-positive cells quantified in the SGZ area (0.36 mm^2^). The values are mean ± SD from 5 animals per experimental group. Two-way ANOVA tests indicate significant differences between investigated groups, *** *p* < 0.001, **** *p* < 0.0001 (effect of ischemia insult) and ^+^ *p* < 0.05; ^++++^ *p* < 0.0001 (effect of TSA treatment) .Abbreviations: ipsi—ipsilateral, contra— contralateral.

The upper panel of Figure 4 illustrates a typical picture of sham-control brains at postnatal day 21 (P21). As it can be seen, BrdU-immunostaining was most pronounced in the DG subgranular zone (SGZ) with the greatest number of cells expressing microtubule associated protein (DCX). In the HI group, the number of BrdU/DCX positive cells was markedly reduced (by about 35%) in the ipsilateral (hypoxic-ischemic) side, compared with sham-controls (mean counts 39 vs. 60, respectively; *p* < 0.0001). Treatment with TSA directly after the HI onset increased BrdU/DCX cells to the control value (mean counts 39 vs. 71, in HI and HI+TSA groups respectively, *p* < 0.0001). In contrast, HI did not affect the number of cells in the contralateral DG. However, we observed that TSA administration elevated the number of newly generated neuroblasts also in the contralateral hemispheres (mean counts 68 vs. 79, *p* < 0,05). Moreover, after HDACi administration, the number of immature neurons detected in this side was higher than in sham-control treated with TSA (71 vs. 63; *p* < 0.001). 

**Figure 5:**
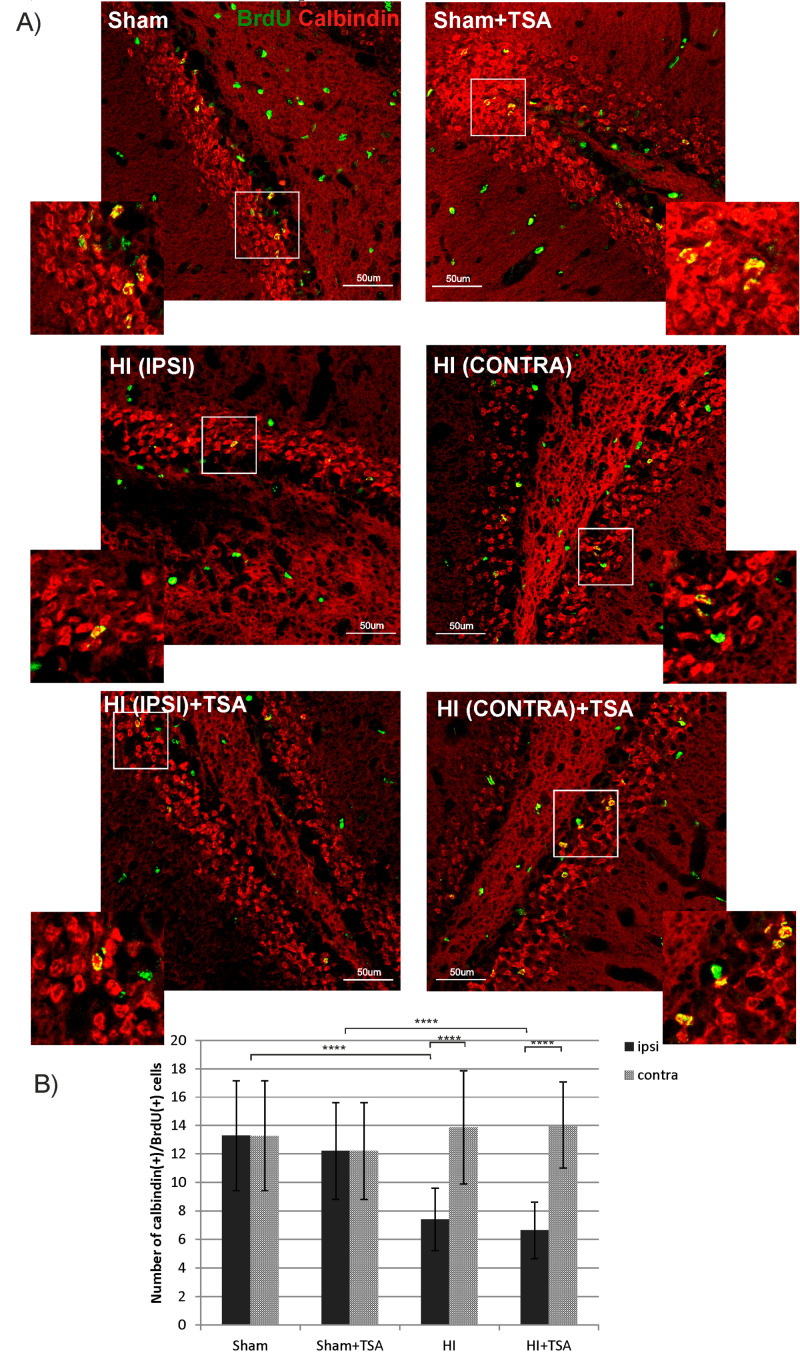
TSA does not affect the number of newborn granule neurons in the DG after HI. (**A**) The confocal photomicrographs show newly generated granule neurons (BrdU/calbindin positive) in the SGZ of sham-control and HI animals D28 with or without TSA treatment. Enlargements present areas marked in rectangles. Scale bar 50 µm. (**B**) The graph presents the number of BrdU/calbindin-positive cells quantified in the SGZ area (0.36 mm^2^). The values are mean ± SD from 5 animals per experimental group. One-way ANOVA tests indicate significant differences between investigated groups, **** *p* < 0.0001. Abbreviations: ipsi—ipsilateral, contra—contralateral.

To address the question of whether new neuroblasts detected in the subgranular zone of DG will develop to mature neurons, we quantified the cells double stained with BrdU and calbindin—a marker of granule neuronal cells at postnatal day 35 (28 days after HI). Quantified results are shown in Figure 5. The number of BrdU/calbindin-positive cells decreased after ischemia in the DG region of the ipsilateral hemisphere compared with sham-controls (7 vs. 13; *p* < 0.0001), without significant effect noticed contralaterally. Post-insult treatment with TSA had no effect on the population of newly generated granule neurons neither in ipsilateral nor contralateral side.

**Figure 6:**
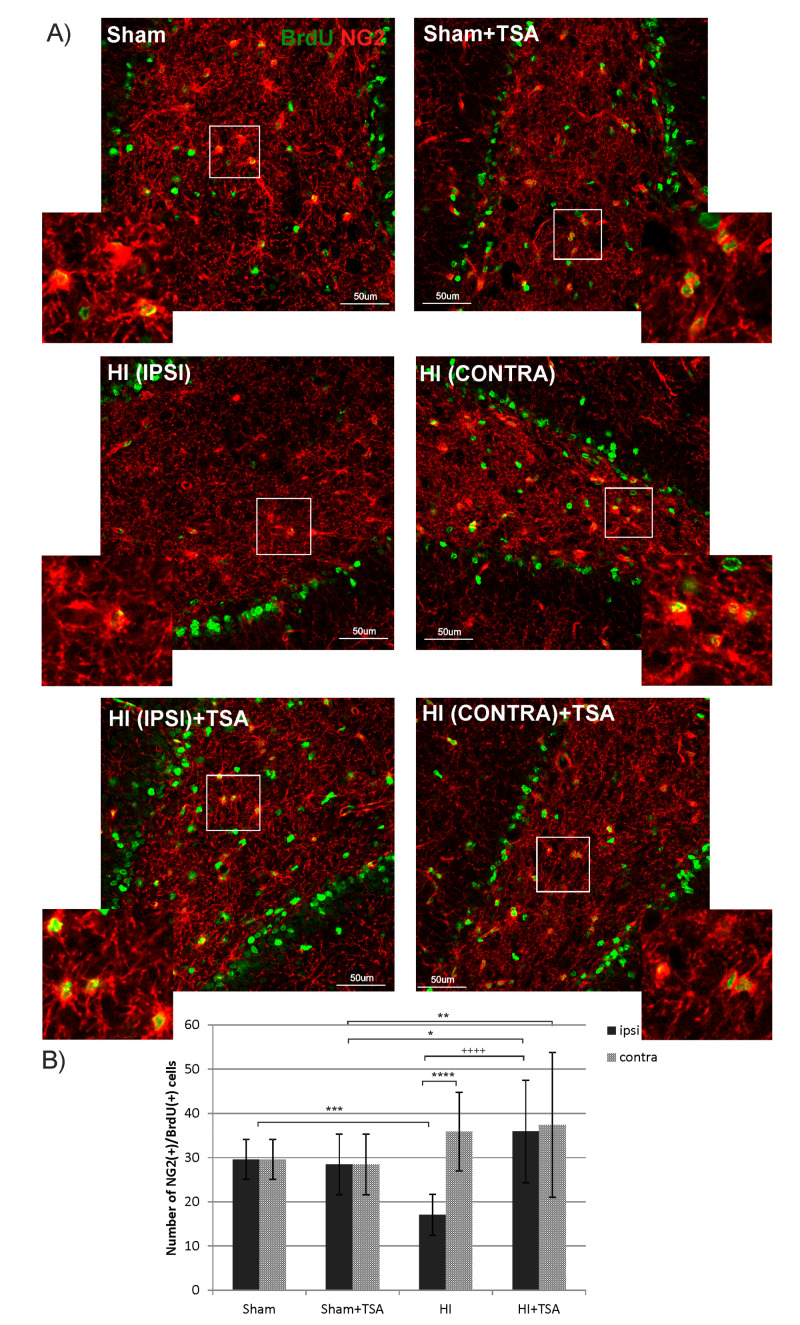
TSA stimulates oligodendrocyte precursor cell proliferation in the DG of the rat hippocampus after HI. (**A**) The confocal photomicrographs show double-labeled (BrdU/NG2-positive) oligodendrocyte precursor cells in the DG of sham-control and HI animals D14 with or without TSA treatment. Enlargements present areas marked in rectangles. Scale bar 50 µm. (**B**) The graph shows the number of BrdU/NG2-positive cells quantified in the DG area (0.36 mm^2^). The values are mean ± SD from 5 animals per experimental group. Two-way ANOVA tests indicate significant differences between investigated groups, * *p* < 0.05; ** *p* < 0.01, *** *p* < 0.001, **** *p* < 0.0001 (effect of HI insult), ^++++^ *p* < 0.0001 (effect of TSA treatment). Abbreviations: ipsi—ipsilateral, contra—contralateral.

**Figure 7:**
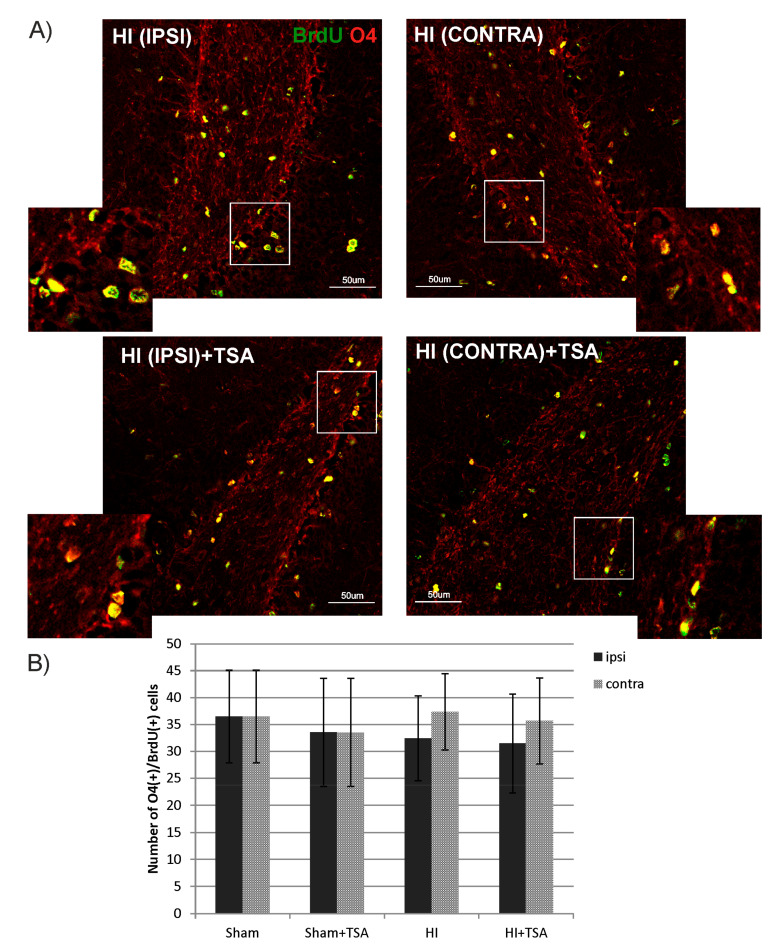
TSA does not affect the number of non-myelinating oligodendrocytes in DG after HI. (**A**) The confocal photomicrographs show double-labeled (BrdU/O4-positive) cells in DG of HI animals D28 with or without TSA treatment. Enlargements present areas marked in rectangles. Scale bar 50 µm. (**B**) The graph shows the number of BrdU/O4 labeled cells quantified in the DG area (0.36 mm^2^). Values represent mean ± SD of five animals per experimental group. Two-way ANOVA tests did not indicate significant differences in the number of BrdU/O4-positive cells between the investigated groups. Abbreviations: ipsi—ipsilateral, contra—contralateral.

To examine whether TSA influences oligodendrogenesis within the DG area, we performed double immunofluorescent staining with BrdU and markers specific for cells in different developmental stages: NG2 to identify oligodendrocyte precursor cells (OPCs) and O4 for more mature oligodendrocytes. The statistical analysis of the data reveals a reduced number of BrdU/NG2-positive cells 14 days after HI (P21) in the ipsilateral hemisphere, compared to the sham-control group (17 vs. 30 cells respectively; *p* < 0.001). Treatment with TSA returned the diminished number of progenitors to the control value (Figure 6). In contrast to the action of TSA on OPCs, neither ischemia nor ischemia treated with TSA changed the number of more mature, however, still non-myelinating oligodendrocytes—BrdU/O4+, analyzed at 4 weeks (P35) after HI induction (Figure 7).

### 2.3. Contribution of Neurotrophic Growth Factors to TSA-Induced Neurogenesis

To investigate the molecular mechanism by which TSA enhanced the generation of neuroblasts and OPCs after neonatal hypoxia-ischemia, we evaluated the expression of neurotrophic growth factors that are involved in neuronal survival and neurogenesis stimulation. We focused our attention on brain derived neurotrophic factor (BDNF), nerve growth factor (NGF), and glia-derived nerve factor (GDNF). 

**Figure 8:**
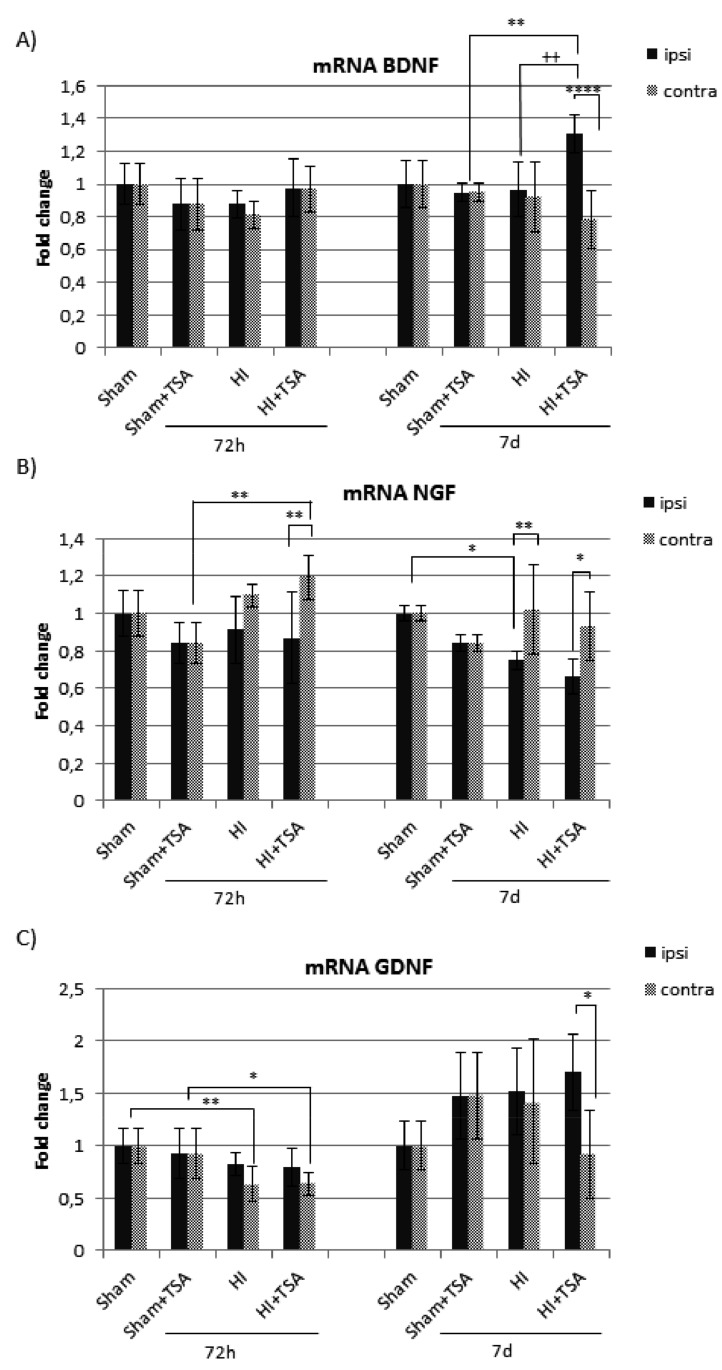
Effect of TSA on mRNA expression of endogenous neurotrophic factors in the brain hemispheres after neonatal hypoxia-ischemia. Real-time RT-PCR was used to quantify neurotrophic factors: (**A**) (brain-derived neurotrophic factor (BDNF), (**B**) nerve growth factor (NGF) and (**C**) glial cell line-derived neurotrophic factor (GDNF)) gene expression in the rat brains 72 h and 7 days after hypoxic-ischemic insult. The fold change of the relative mRNA expression of each studied gene was calculated with the 2−ΔΔCT method. The data represent the normalized target gene amount relative to control, which is considered 1. Note the increase in BDNF mRNA expression in the ipsilateral hemisphere after TSA treatment at 7 days of recovery. The values are mean ± SD from 5 animals per group and time point assessed in triplicates. Two-way ANOVA tests indicate significant differences between investigated groups, * *p* < 0.05,** *p* < 0.01,**** *p* < 0.0001 (effect of HI insult), ^++^ *p* < 0.01 (effect of TSA treatment). Abbreviations: ipsi -ipsilateral, contra-contralateral.

The expression of BDNF, NGF and GDNF mRNA was evaluated by real-time PCR at 3 and 7 days after hypoxia-ischemia. Our study shows a significant effect of HI only in the case of gene coding NGF, compared with the matching sham-controls (Figure 8B). The remarkable reduction of mRNA is seen in the ipsilateral hemisphere 7 days after HI, independent of the TSA treatment. We also observed a modest but statistically significant reduction of GDNF mRNA but only in the hypoxic/contralateral hemisphere, both at 3 and 7 days after the insult (Figure 8C). Although hypoxic-ischemic injury did not affect the expression of BDNF mRNA, the presence of TSA stimulated BDNF gene expression in the damaged hemisphere 7 days after the insult (*p* < 0.01) (Figure 8A). In contrast, no effect of TSA was noted in the case of NGF and GDNF mRNA. 

**Figure 9:**
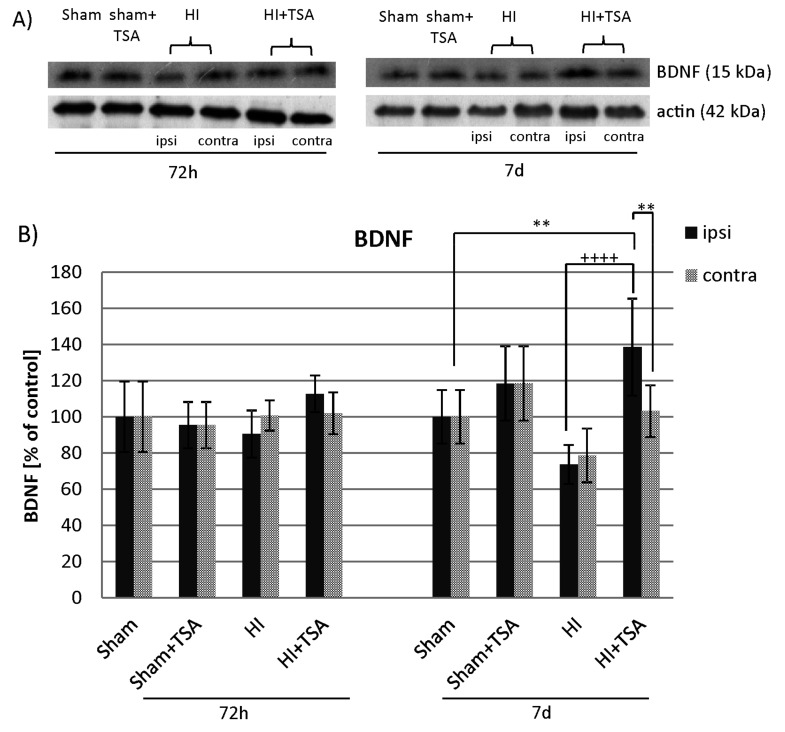
Effect of TSA on the expression of BDNF protein in the brain hemispheres after HI. (**A**) Representative immunoblots of BDNF protein level. The intensity of each band was quantified and normalized in relation to actin. (**B**) The bar graphs represent the statistical analysis of immunoreactive bands estimated in indicated experimental groups: sham-control (Sham), TSA-treated sham-control (Sham+TSA), hypoxia-ischemia (HI), TSA-treated hypoxia-ischemia (HI+TSA). Note that TSA application increases the level of BDNF protein at 7 days of recovery. The values are mean ± SD from 5 animals per group and time point assessed in 3 independent experiments. Two-way ANOVA tests indicate significant differences between investigated groups, ** *p* < 0.01, ^++++^ *p* < 0.0001 (effect of TSA treatment). Abbreviations: ipsi—ipsilateral, contra—contralateral.

To investigate if the effect of TSA on BDNF mRNA expression translates on the BDNF protein level, we performed Western Blot experiments at 3 and 7 days after hypoxia-ischemia induction. As shown on Figure 9, TSA treatment led to a remarkable increase in BDNF protein expression in the ipsilateral hemisphere 7d after HI (*p* < 0.001 ipsilateral vs. ipsilateral with TSA). After 72h post-injury, the expression of BDNF protein presented almost the same value in all investigated groups.

### 2.4. The Effect of TSA on TrkB Receptor

**Figure 10:**
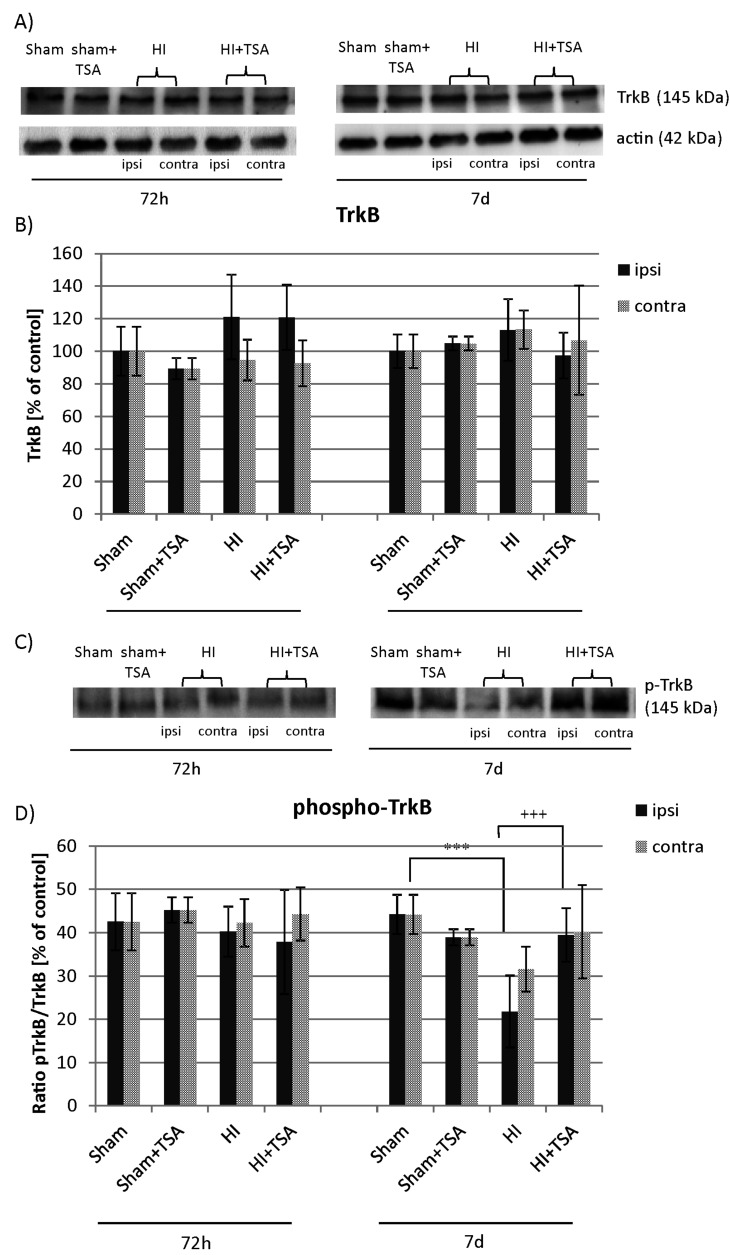
Effect of TSA on the expression of TrkB and phospho-TrkB in the brain hemispheres after HI. Representative immunoblots of total TrkB protein (**A**) and phosphorylated TrkB (**C**) level. The intensity of each band was quantified and normalized in relation to β-actin. The bar graphs (**B** and **D**) represent statistical analysis of immunoreactive bands estimated in indicated experimental groups: sham-control (Sham), TSA-treated sham-control (Sham+TSA), hypoxia-ischemia (HI), TSA-treated hypoxia-ischemia (HI+TSA). Note that TSA application increases the level of TrkB phosphorylation at 7 days of recovery. The values are mean ± SD from 5 animals per group and time point assessed in 3 independent experiments. Two-way ANOVA tests indicate significant differences between investigated groups, *** *p* < 0.001 (effect of HI insult), ^+++^ *p* < 0.001 (effect of TSA treatment).. Abbreviations: ipsi—ipsilateral, contra—contralateral.

Many studies have shown that the neurophysiological effects of BDNF are mediated by the activation of its cell-surface receptor TrkB. In the present study, we performed Western Blotting to assess the protein level of the TrkB receptor as well as its activation/phosphorylation at 3 and 7 days after hypoxia-ischemia induction. The immunoreactivity measurements indicate that hypoxic-ischemic insult neither with nor without TSA treatment affect the expression of TrkB native protein. However, a clear reduction in phosphorylated receptor expression to about 55% of the value characteristic for sham-control (*p* < 0.001) was seen in the injured hemisphere 7 days after HI. The application of TSA after HI insult blocked the effect of HI and kept the immunoreactivity of phospho-TrkB in the ipsilateral side on the control level (*p* < 0.001, ipsilateral vs. ipsilateral with TSA) (Figure 10). 

### 2.5. The Influence of TSA on Transcription Factor Phospho-CREB 

**Figure 11:**
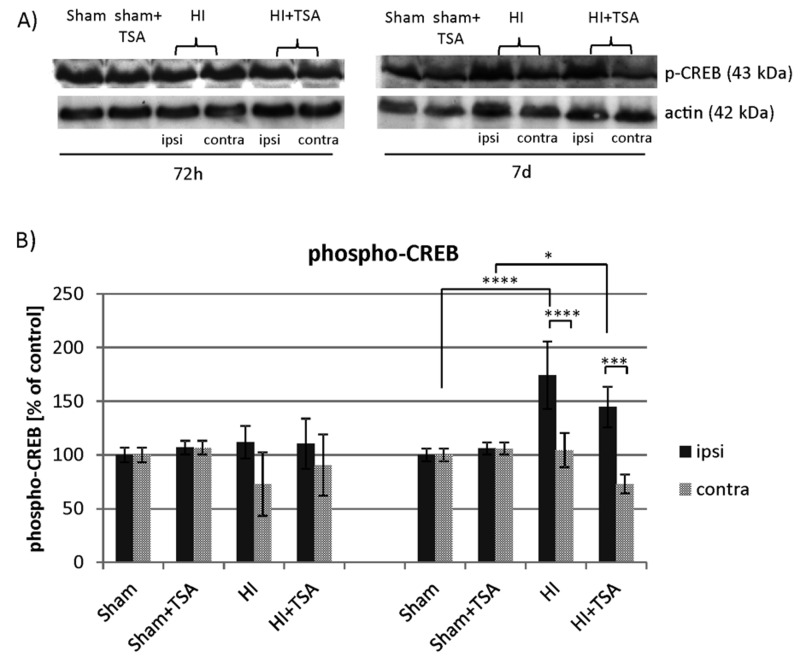
TSA has no effect on CREB phosphorylation after neonatal hypoxia-ischemia. (**A**) Representative immunoblot of phospho-CREB protein level after TSA treatment in sham-control and HI animals. The intensity of each band was quantified and normalized in relation to actin. (**B**) The bar graphs represent the statistical analysis of immunoreactive bands estimated in four experimental groups: sham-control (Sham), TSA-treated sham-control (Sham+TSA), hypoxia-ischemia (HI), TSA-treated hypoxia-ischemia (HI+TSA). Note the increase in CREB phosphorylation in ipsilateral hemisphere 7 days after HI, regardless of TSA application. The values are mean ± SD from 5 animals per group and time point assessed in 3 independent experiments. Two-way ANOVA tests indicate significant differences between investigated groups, * *p* < 0.05, *** *p* < 0.001, **** *p* < 0.0001. Abbreviations: ipsi—ipsilateral, contra—contralateral.

Phospho-CREB is an important factor downstream of the BDNF-TrkB signaling pathway and has been shown to be involved in neurogenesis in the DG. Using a Western Blot assay we examined phospho-CREB (Ser 133) immunoreactivity (Figure 11). The densitometric analysis of obtained immunoblots revealed significant changes in the phospho-CREB level at 7 days after the insult in the injured hemisphere, when the value reached 174% of sham-control (*p* < 0.0001). Treatment with TSA maintained the phosphorylation level at a similar, elevated value (144% of sham-control). 

## 3. Discussion 

Several studies performed in the past years have shown the neuroprotective effect of histone deacetylase inhibitors (VPA, TSA, SB, SAHA) in both in vitro and in vivo experimental settings in adult rodents [13,14,28]. The neuroprotective efficacy of these agents has been mainly explored in adult models of brain ischemia. It was demonstrated that the action of HDACis was associated with an expanded population of proliferating cells and neurogenesis. It is worth pointing out that, to date, only a few papers described the consequences of histone deacetylase inhibitor treatment in the immature brain and those addressing the effect of these agents remained particularly limited [18,19]. Hence, in the present study, we used a rat model of neonatal hypoxia-ischemia to determine whether TSA, a potent broad HDAC inhibitor, will stimulate endogenous neurogenesis in the DG of the ipsilateral damaged side of the maturing brain. Our study provides evidence that TSA treatment may be able to restore the population of immature neurons, neuroblasts (identified by BrdU/DCX staining), in the subgranular layer of the hippocampal dentate gyrus ipsilateral to lesioning two weeks post injury. However, in spite of the enhanced generation of the new immature neurons, they do not develop to the more mature phenotype of granule neurons (BrdU/calbindin positive), at least not after four weeks. This finding remains in agreement with our previous report showing a reduced number of full-grown neurons in the ipsilateral hemisphere at 28 days after HI, regardless of treatment with sodium butyrate (SB), another inhibitor of histone deacetylases [22]. However, in the present study, we did not examine neurogenesis in the SVZ, the second neurogenic region of the brain. Our previous results [23] have shown that treatment with sodium butyrate in the same model of neonatal HI, increases the number of mature, NeuN (+)/BrdU (+) neurons in the SVZ, but not in the DG region. On the other hand, increased neurogenesis after SB did not translate to improving the neurobehavioral performance of tested animals [22].

At present, the molecular signals responsible for the incomplete neurogenesis after TSA treatment are not clear. It may be due to the depletion of newly generated cells as a contributing factor, despite the seemingly robust production of neuroblasts, based on DCX immunostaining, or that the injured SGZ environment at this developmental stage is not conducive to the maturation and survival of newly formed neurons, since there may be a deficiency of trophic support, in agreement with other published data [29,30,31]. In accordance with this, it is known that, after HI insult, the cellular and molecular structure of the neurovascular niche undergoes alterations [32,33,34]. Therefore, although cell proliferation is preserved in this neurogenic area, detrimental changes in protein expression in the brain after ischemia can lead to the impairment of neuronal fate. From the above, it follows that, in the present experimental conditions, the endogenous neurogenesis is not sufficient for compensation of lost neuronal circuits. 

It should be noted that although the enhancement of endogenous total proliferation of cells has already been demonstrated in rodent models of brain ischemia after HDACis administration [35], the unchanged number of BrdU-positive cells in the hippocampus at 14 and 28 days after HI have shown that the treatment with TSA did not expand the total population of proliferating cells in the hippocampal dentate gyrus of maturing brain, compared with age-matched sham animals. The unchanged intensity of proliferation was also observed by Qiu et al. [36] and Scheepens at al. [37] in mice subjected to HI. It is possible that high level of plasticity and the degree of neurogenesis in the immature brain cannot be upregulated any further by epigenetic response. The identification of factors that are responsible for differential response between animals of the same species at different maturational stages will likely reveal critical mechanisms that control injury-induced stem cell proliferation and neurogenesis. 

Neuroprotection with TSA in neonatal HI models is also associated with a reduction in oligodendrocyte progenitor cell death. We showed that, after TSA application, the number of OPCs (BrdU/NG2+) in ipsilateral hippocampi 2 weeks after the insult returned to the control level, restoring the physiological pool of progenitors, which could be conducive for sustaining homeostasis. NG2+ cells exhibit the ability to proliferate throughout the life span and serve as a reservoir of glial progenitors for deriving myelinating cells [38]. The progress in the differentiation of the oligodendrocytes towards the O4 antigen, attributed to a more advanced stage of oligodendrocyte maturation, was noticed at 28 days post injury. Our data shows that the number of these cells did not differ significantly between investigated experimental groups, independent of the presence of inhibitor. It might be related to the active gliogenesis process ongoing strongly during the perinatal period as well as the first postnatal weeks in the rat’s brain even after neonatal hypoxia-ischemia. It is worth pointing out that oligodendrocyte progenitors are able to secrete factors that modify local microenvironment and neighbouring cell reactions and can potentially take part in the initiation of endogenous restorative processes [39,40,41]. This presumption might explain the observation that the number of mature oligodendrocytes is often undisturbed in pathological conditions. Despite the fact that, in our present work, we did not investigate the generation of myelinating oligodendrocytes, an unchanged level of myelin basic protein (MBP) and myelin proteolipid protein (PLP) after hypoxia-ischemia found in our preceding studies highlighted the efficiency of the ongoing myelination [42]. 

Of note, some reports indicate that oligodendrocyte differentiation is regulated by HDAC. Thus, it might be speculated that blockage of histone deacetylase activity by an inhibitor during the first days after birth suppresses oligodendrocyte maturation, and by this leads to hypomyelination [43]. However, it occurred that the inhibition of oligodendrocyte differentiation by HDACi is transient and occurs only during first 2 postnatal weeks and as such does not impair the differentiation process. In contrast, the data of Fleiss using Olig-2 marker of oligodendrocytes indicate that TSA treatment in neonates after LPS/HI does not affect the regulatory mechanism governing oligodendrocyte maturation [20]. 

Under physiological conditions neural stem cells (NSC) in the SGZ region differentiate mainly into neurons but some of them can generate new astrocytes [44]. In post-ischemic tissue, neuronal maturation is delayed and NSCs’ differentiation is promoted towards glial cells [45]. In the present study, we did not examine generation of new astrocytes after HI and TSA treatment; however, it is highly possible that hypoxia-ischemia and/or injection of TSA causes the generation of new astrocytes in DG region. The reduced number of BrdU(+)/DCX(+) and BrdU (+)/NG2(+) cells in the ipsilateral hemisphere at 14 days after HI, with a lack of overall difference in the number of BrdU positive cells, can support this assumption. It is possible that an unchanged level of cell proliferation with a simultaneous decrease in the number of new neuroblasts and oligodendrocyte progenitors after HI may be caused by an enhanced number of newly born astrocytes. Our previous experiments showed intensive astrogliosis mainly in the cortex of ipsilateral hemisphere at 6 days after HI, manifested by an increased number of hypertrophic GFAP (+) cells. The treatment with sodium butyrate, another HDAC inhibitor, markedly increased the expression of GFAP [46]. It is very likely that TSA has a similar effect.

Although many reports show a positive effect of HDACi treatment in various brain injuries, the mechanism regulating this response remains unclear. A body of work postulated that the action of HDACi after ischemia is coupled with the up-regulation of histone H3 or H4 acetylation, a virtue of HDAC inhibitory activity [13,14,20]. It should be pointed out that chromatin remodeling via HDAC inhibition produces resistance to multiple insults in the CNS [47]. Therefore, it is possible that the effects of HDAC inhibition were associated with their ability to protect cells arisen after HI from premature death. However, in contrast to others, the positive effect of TSA on the generation of new neuroblasts and OPCs after neonatal HI does not appear to be mediated via acetylation of histones, as was demonstrated by Western Blot assay data. Hence, the neurogenic response to TSA treatment might likely represent direct or indirect effects of non-histone HDACi targets, including transcription factors, cytoskeletal proteins, molecular chaperons, and nuclear import factors [15,17,48]. Indeed, despite the fact that TSA does not alter histone acetylation, it had an effect on alpha-tubulin acetylation, which also an important target of deacetylase inhibitors. In addition to regulating microtubule architecture, tubulin acetylation is engaged in various cellular processes, including facilitation of axonal transport. The significant decrease in acetylated tubulin observed after hypoxic-ischemic injury in the ipsilateral hippocampus might lead to axonal cytoskeletal breakdown and disturbances in axonal transport, an established feature of ischemic brain injury [49,50,51]. After TSA administration, the acetylation of alpha-tubulin returned to the control level, in agreement with the already reported effect of this agent in vitro as well as in vivo on axonal flow rescue [52]. Speculatively, the action of TSA might be linked with increased vesicular transport and thus the subsequent secretion of trophic factors (for example, BDNF, NGF, and GDNF) as was demonstrated in sensory and motor neurons [53]. 

Of interest, the role of trophic factors, together with their corresponding receptors, in the generation and survival of newborn cells after brain ischemia and trauma, has been documented [54,55,56,57,58,59]. Hence, interventions using HDACis to restore axonal transport seem to be a crucial approach to reverse degenerative effects [60,61]. Despite the reported link between NGF and GDNF signaling and proper proliferation, the differentiation and survival of newly generated cells [62,63] to our surprise in the current study TSA treatment failed to increase the gene expression of GDNF as well as NGF in the ipsilateral side after HI. On the contrary, we detected the significant up-regulation of BDNF mRNA in the damaged hemisphere 7 days after HI. Given this above dissimilar response of neurotrophic factors, it might be speculated that neuroprotective effects depend upon developmental and contextual milieu. It should be pointed out that the timing of the BDNF protein elevation found after SB treatment in our earlier study correlated well with the appearance of new neuroblasts in the SGZ, which strongly supports the implication of this factor in neurogenesis. The role of BDNF could be reinforced by data reported by Yasuda et al. [64], showing that HDAC inhibition activated BDNF promoter IV and increased BDNF mRNA levels in dissociated rat cortical neurons. The key role of BDNF in neurogenesis has been additionally verified by the administration of this neurotrophin into the lateral ventricle of adult rats, which leads to the generation of new neurons [54], while the BDNF knock-out suppresses the generation of new neural cells in adult hippocampus [65]. 

It is postulated that the beneficial effect of HDACi on the proliferation and differentiation of neuronal cells in the adult rodents after middle cerebral artery occlusion (MCAO) [35,66] is mediated, at least partially, by the binding of BDNF to TrkB receptor and the activation of the downstream mediators of the BDNF-TrkB pathway. One of the most interesting results in the present study, which may support the role of BDNF-TrkB signaling in TSA-induced neurogenesis, is the decreased level of the activated/phosphorylated TrkB receptor after HI, which returns to control value after TSA treatment. The activation of the TrkB receptor in in vitro and in vivo studies of rodent mature brain is coupled with the activation of appropriate downstream pathways that are connected to the phosphorylation of transcription factors such as CREB [35,64,67]. The activation of CREB through Ser 133 phosphorylation has been shown to mediate neurogenesis in the adult DG after focal ischemia in rodents [68]. In addition, phospho-CREB directly regulates the expression of BDNF, which also enhances the survival and differentiation of progenitor cells in vitro and increases the number of newborn cells in vivo [69,70]. The elevation of phospho-CREB in damaged (hypoxic-ischemic) hemisphere one week after injury induction without or with TSA treatment may act as a protective mechanism associated with the enhancement of pro-survival gene expression. The above speculation remains in agreement with observed stimulation of CREB phosphorylation in adult animals after brain ischemia. [71,72,73]. However, on the basis of our results, it seems that TSA is not involved in the neurogenic effect of CREB activation. 

TSA, as well as other HDAC inhibitors, presents a majority of unspecific effects and it is not possible to identify the key pathway responsible for neurogenesis. Convincing evidence reveals that HDACis treatment after experimental brain ischemia in adult animals is neuroprotective and improves neuropathological outcome, which may result from the anti-inflammatory proprieties of these agents [13,35]. However, in the present study, we did not examine the influence of TSA treatment on inflammatory reaction, it is possible that this inhibitor may reduce inflammation in the brain after neonatal HI. As demonstrated in our previous study, another HDAC inhibitor—SB—robustly diminished the generation of microglial cells in the ipsilateral hemisphere 14 days after HI injury. Moreover, SB facilitates the conversion of pro-inflammatory M1 microglia phenotype to anti-inflammatory M2 phenotype and consequently prevents tissue damage and may enhance endogenous neurogenesis [23,46].

## 4. Material and Methods

### 4.1. Experimental Neonatal Hypoxia-Ischemia

All experiments conducted on animals were approved by the 4th Local Ethics Committee for Animal Experimentation in Warsaw, Poland (approval date: 22 May 2015, permit no. 83/2015), according to EU Directive 2010/63/EU. Neonatal hypoxia-ischemia was induced in postnatal-day-7 (P7) Wistar rats, weighing 16 ± 1 g, by a permanent unilateral common carotid artery ligation, followed by systemic hypoxia [74]. In our previous studies, it was explored if there is a sexually dimorphic response to HI treatment [23]. As we did not notice any significant differences between male and female rats in the previous experiments, pups of either sex were used. In brief, rat pups were anesthetized with isoflurane (4% induction, 2.0% maintenance) and the left common carotid artery was double ligated with surgical silk and cut between ligatures. The incision was sutured, and the wound was treated with lignocaine as analgesic. Sham-control animals underwent the same surgical procedure without the ligation of the artery. After the surgical procedure, rat pups were returned to their dam for 1 h of recovery. Afterwards, the animals were placed in a chamber (35 °C) and subjected to a mixture of 7.6% oxygen in nitrogen for 1 h to induce hypoxia. The surgical operation is simple, brief, and the whole procedure associates with very low animal mortality. In this model, the hypoxic-ischemic lesion occurred in the cerebral hemisphere on the side of the ligated artery (ipsilateral), while the opposite hemisphere (contralateral) was treated as an internal control. Additional controls were the brains of sham-operated animals. 

After the entire procedure, the pups were returned to their home cage (type 1291H; 425 mm × 266 mm × 185 mm) with the TierWohl Super as a bedding and enriched with Lignocel Nesting Large material. Animals were housed at 22 ± 2 °C, 55 ± 5% humidity, 12/12 h light/dark cycle and fed by their dams. At postnatal day 21 (PND 21), rats were separated from their mothers and at least two litter mates were housed in one cage with free access to water and food ad libitum. During the experiment, animal mortality did not occur outside of planned endpoint.

**Figure 12:**
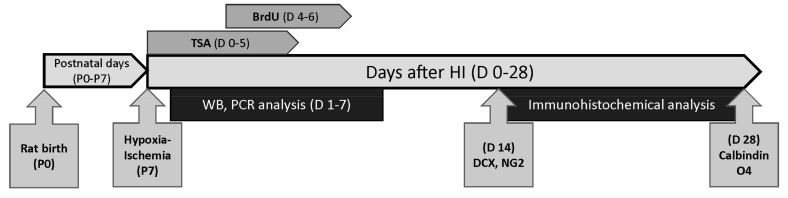
Diagram of experiment design in the present studyNeonatal hypoxia-ischemia was induced in 7-day-old (P7) Wistar rat pups. Sham-control and HI animals were subcutaneously injected with TSA (0.2 mg/kg body weight) or vehicle (10% DMSO in saline) at the same volume starting immediately after hypoxic exposure and lasting for 5 consecutive days (D 0–5). All animals dedicated to immunohistochemical studies received twice-daily intraperitoneal injections of BrdU (4–6 day after HI (D 4–6)). Animals in this group were perfused transcardially with 4% PFA 14 (D14) or 28 (D28) days after HI, and immunohistochemical analysis was performed. Western lot and PCR analysis were performed on non-perfused brains (1, 3 7 days after the insult (D 1–7)).

Rats from each litter were randomly assigned to 4 experimental groups (5 rats per group and time point): (1) control sham-operated animals (Sham) (2) control sham-operated animals treated with TSA (Sham+TSA), (3) animals after hypoxia-ischemia (HI), (4) animals after hypoxia-ischemia treated with TSA (HI+TSA). Animals were sacrificed at specific time points: 1, 3, 7, 14 or 28 days after the injury. For the immunohistochemical studies, 40 rats—18 females and 22 males—were used (4 groups and 2 time points: Sham (3F, 7M); Sham+ TSA (4F, 6M); HI (6F, 4M); HI+TSA (5F, 5M)), and for other analysis (PCR and Western blot) we used 60 rats—28 females (F) and 32 males (M)—(4 groups and 3 time points: 6 M and 9 F in Sham groups; 10 M and 5 F in Sham+TSA groups; 7 M and 8 F in HI groups and 9 M and 6 F in HI+TSA groups). The animal experiments are summarized in Figure 12.

### 4.2. Drug Administration and Bromodeoxyuridine Labeling

#### 4.2.1. Drug Treatment 

Sham-control and HI rats were subcutaneously injected with Trichostatin A (TSA, Sigma-Aldrich, St. Louis, MO, USA) (0.2 mg/kg body weight) or vehicle (10% DMSO in saline) at the same volume starting immediately after hypoxic exposure and lasting 5 consecutive days (one dose every 24 h). The dose of TSA was comparable with those used in rats [13,66].

#### 4.2.2. Bromodeoxyuridine Labeling 

Animals intended for immunohistochemical studies received intraperitoneal injections of 5-bromo-2-deoxyuridine (BrdU, Sigma-Aldrich, 50 mg/kg dissolved in sterile 0.9% NaCl plus 0.007 N NaOH) at 12-h intervals for 3 consecutive days starting on the 4th day after hypoxia-ischemia induction. The time frame of BrdU administration was determined in our previous studies [22,23]. This procedure was used to determine the phenotype of the newborn cells in immunohistochemical studies. 

### 4.3. Tissue Preparation

Rats surviving 14 or 28 days after HI or aged-matched controls were deeply anesthetized with intraperitoneal injections of 100 mg/kg body weight ketamine combined with 10 mg/kg body weight xylazine and perfused transcardially first with phosphate-buffered saline (PBS) and then with fixative solution of 4% paraformaldehyde in PBS (pH 7.4). Afterwards, the brains were dissected from the scull, post-fixed for 3 h at 4 °C in the same fixative solution, cryoprotected overnight in 30% sucrose solution and finally frozen on dry ice. Coronal cryostat brain sections (30 µm thick) were cut at the level of the rostral/central part of the hippocampi (from Bregma−2.80 mm Lambda 4.80 mm to Bregma 3.40 mm Lambda 4.20 mm) in serial order to create 10 series sections and used for immunohistochemical studies. 

Biochemical and PCR analysis were performed on non-perfused brains. The rats were deeply anesthetized with ketamine and xylazine (as described above) and decapitated 1, 3 and 7 days after HI, the brains were isolated, and dissected on 2 hemispheres: ipsilateral (injured/hypoxic-ischemic) and contralateral(non-injured, hypoxic only). Afterwards, the tissue was immediately frozen on dry ice and stored at −80 °C until use. 

### 4.4. Immunohistochemical Staining

The following antibodies were used for immunohistochemical staining (, source, catalogue number and final dilution): sheep polyclonal anti-BrdU (Abcam, Cambridge, UK, cat. no ab1893, 1:500), mouse monoclonal anti-BrdU (Santa Cruz Biotechnology, Dallas, TX, USA, cat. no sc-32323, 1:100), rabbit polyclonal anti-Doublecortin (DCX; Cell Signaling, Danvers, MA, USA, cat. no 4604, 1:200), rabbit monoclonal anti-Calbindin (Cell Signaling, Danvers, MA, USA, cat. no 13176, 1:200), rabbit polyclonal anti-NG2 Chondroitin Sulfate proteoglycan (Milipore, Burlington, MA, USA, cat. no AB5320, 1:200), mouse monoclonal anti- oligodendrocyte marker (O4; Milipore, Burlington, MA, USA, cat. no MAB345, 1:200).

Double fluorescent immunohistochemistry was performed on free-floating sections. To detect BrdU incorporation, sections were incubated for 1 h with 2N HCl at 37 °C for DNA denaturation. Afterwards, brain sections were rinsed for 15 min in 0.1 M sodium tetraborate (pH 8.5) at room temperature. After blocking for unspecific reactivity with 10% normal goat or donkey serum (Sigma-Aldrich, St. Louis, MO, USA) in PBS containing 0.25% Triton X-100 (Sigma-Aldrich St. Louis, MO, USA) for 1h, sections were incubated with anti-BrdU overnight at 4 °C. Then, sections were washed (3 × 5 min in PBS) and stained with appropriate secondary fluorescein isothiocyanate (FITC)-conjugated (Invitrogen, Waltham, MA, USA, 1:500) antibodies for 1 h at room temperature. 

The differentiation of BrdU-positive cells was monitored with markers labeling neurons or oligodendrocytes at various stages of maturation. After BrdU staining, the brain sections were incubated with appropriate primary antibodies (anti-DCX, anti-calbindin, anti-NG2, anti-O4) overnight at 4 °C. Afterwards, the sections were rinsed in PBS (3 × 5min) and stained in the dark with secondary appropriate Cy3-conjugated antibodies for 60 min. After a final washing in PBS (3 × 5min), sections were mounted on slides using Dako fluorescent mounting medium. 

Labeling was verified using a confocal laser scanning microscope (LSM 780, Carl Zeiss, Germany) with ZEN software. A helium-neon laser (543 nm) was utilized in the excitation of Alexa Fluor 546 (Cy3), while an argon laser (488 nm) was applied in the excitation of FITC. 

The number of double-stained cells was manually counted in an average of five brain sections per animal in a 1.44 mm^2^ area of the hippocampal DG utilizing ImageJ 1.46 software. All analyzed confocal images were coded, and the experimenter who counted double-stained cells was unaware of the sample source.

### 4.5. Quantitative Polymerase Chain Reaction (Real-Time PCR) 

The gene expression of neurotrophins (BDNF, NGF, GDNF) was assessed in brain hemispheres obtained from rats 3 and 7 days after HI. Total RNA was isolated with TRIzol Reagent (Thermo Fisher Scientific, Waltham, MA, USA) and the quality and concentration of RNA was verified by spectrophotometry with the Nanodrop™ apparatus. The samples containing 1 μg of total RNA were reverse transcripted using a High-Capacity RNA-to-cDNA Kit (Thermo Fisher Scientific, Waltham, MA, USA) according to the manufacturer’s instructions.

Quantitative real-time PCR analyses of cDNA samples (300 ng) with Fast SYBR Green Master Mix (Thermo Fisher Scientific, Waltham, MA, USA) and specific primers (Table 1) were performed in a 7500 Fast Real-Time PCR System (Applied Biosystem, Waltham, MA, USA). The reaction parameters were as follow: (1) holding stage: 20 s at 95 °C; (2) cycling stage (40×): 3 s at 95 °C, 30 s at 60 °C; (3) melt curves stage: 15 s at 95 °C, 1 min at 60 °C, increase 0.5 °C per cycle for 10 s from 60 °C to 95 °C. The fluorescence signal from a specific transcript was normalized against that of the reference gene (SDHA), and threshold cycle values (Δ*C*t) were quantified as fold changes by the 2 ^−ΔΔ*C*t^ method.

### 4.6. Western Blot Analysis

The following antibodies (source, catalogue number, and final dilution) were used for analysis: rabbit polyclonal anti acetyl-H3 (Millipore, Burlington, MA, USA, cat. no 06-599, 1:1000), rabbit monoclonal anti acetyl-alpha-Tubulin (Lys40) (Cell Signaling, Danvers, MA, USA, cat. no 5335, 1:1000), mouse monoclonal anti-BDNF (Abcam, Cambridge, UK, cat. no ab203573, 1:500), rabbit polyclonal anti-TrkB (Santa Cruz Biotechnology, Dallas, TX, USA, cat. no sc-12, 1:200), rabbit polyclonal anti-phosphoTrkB (pTyr515) (Thermo Fisher Scientific, Waltham, MA, USA cat. no PA5-36695, 1:200), mouse monoclonal anti-phospho CREB (Ser133), (Millipore, Burlington, MA, USA, cat. no 05-807, 1:500), mouse monoclonal anti-actin (MP Biomedicals, Irvine, CA, USA, cat. no 0869100 MP, 1:500).

The Western Blot procedure was described in our previous publication [23]. Briefly, hemispheres were homogenized in RIPA lysis buffer (10mM Tris-HCl pH 7.5 containing 150 mM NaCl, 1% Nonidet P40, 0.1% SDS, 1% Triton X-100, PMSF 0.1 mg/mL) supplemented with proteinase and phosphatase inhibitors cocktail (Sigma Aldrich, St. Louis, MO, USA, 1:100). Lysates were centrifuged at 13,000× *g* for 10 min at 4 °C. The supernatant was collected and used for analysis of acetyl-alpha-Tubulin, TrkB, pTrkB and p-CREB. Cell pellets were resuspended in RIPA lysis buffer and used for analysis of acetyl-H3. Total protein concentrations (in the supernatant as well as in the pellet solution) were assessed by a Bio-Rad DC^TM^ protein assay kit (Bio-Rad, Hercules, CA, USA). Samples (50 µg protein/per well) were separated by SDS-PAGE and transferred onto nitrocellulose (Amersham^TM^ Protran^TM^ supported 0.45 μm NC), and proteins were detected with specific primary antibodies and then incubated with appropriate horseradish peroxidase-conjugated secondary IgG antibodies (Sigma-Aldrich, St. Louis, MO, USA). Immunoblot signals were visualized using an ECL chemiluminescence kit (GE Healthcare Life Sciences, Marlborough, MA, USA). Actin antibody was used as an internal control for each immunoblotting. The smi-quantitative evaluation of protein levels detected by immunoblotting was performed utilizing LKB Utrascan XL, Program GelScan software. The densitometry values were averaged in all groups and then the densitometry values in the sham-control groups were taken as 100%. The data from indicated experimental groups were presented as percent of control value. 

### 4.7. Statistical Analysis

Statistical analysis of the received data was performed using the GraphPad PRISM 5.0 software. The two-way analysis of variance (ANOVA) followed by the Tukey’s or Sidak’s multiple comparison tests were used. All received data was compared using (a) ischemia surgery and (b) TSA treatment as two independent factors. All values were expressed as mean ± SD. The data were considered significant at *p*-value <0.05.

## 5. Conclusions 

Our results show that TSA, an inhibitor of histone deacetylases, stimulates the generation of new neuroblasts and oligodendrocyte progenitors in the damaged ipsilateral dentate gyrus. We suggest that the neurogenic effect of TSA may be mediated, at least partially, by the BDNF-TrkB signaling pathway. However, in the present experimental conditions, the activation of this pathway is not sufficient to increase the number of new mature granule neurons and oligodendrocytes, which indicates a limited effect of TSA on this neurogenesis stage.

## Figures and Tables

**Table 1:** Primers used in reverse transcription and quantitative real-time RT-PCR analysis.

	Forward Primer Sequence	Reverse Primer Sequence	Product Length (bp)
BDNF	5′-CGGCTGGTGCAGGAAAGCAA-3′	5′-TCAGGTCACACCTGGGGCTG-3′	136
NGF	5′-CCCGAATCCTGTAGAGAGTGG-3′	5′-GACAAAGGTGTGAGTCGTGG-3′	82
GDNF	5′-AAGGTCGCAGAGGCCAGAGG-3′	5′-TCTCGGCCGCTTCACAGGAA-3′	144
SDHA	5′-CCCTGAGCATTGCAGAATC-3′	5′-CATTTGCCTTAATCGGAGGA-3′	60

## Abbreviations

Acetyl-H3	Acetylated Histone H3
BDNF	Brain-derived neurotrophic factor
BrdU	5-bromo-2’-deoxyuridine
CREB	cAMP response element-binding protein
CA1	Cornu Ammonis region 1
contra	Contralateral hemisphere (not injured, hypoxic only)
DCX	Neuronal migration protein doublecortin
GDNF	Glial cell line-derived neurotrophic factor
HDACis	Histone deacetylase inhibitors
HDACs	Histone deacetylases
HI	Hypoxia-ischemia
ipsi	Ipsilateral hemisphere (injured, hypoxic-ischemic)
LPS	Lipopolysaccharide
LSM	Laser Scanning Microscope
MBP	Myelin basic protein
NG2	Nerve/glial antigen 2, Chondroitin sulfate proteoglycan 4
NGF	Nerve growth factor
O4	Late oligodendrocyte progenitor-specific marker
OPCs	Oligodendrocyte progenitor cells
PLP	Proteolipid protein
SB	Sodium butyrate
SGZ	Subgranular zone
Trk	Tropomyosin receptor kinase
TSA	Trichostatin A
VPA	Valproic acid
